# The Lack of Ireland’s Assisted Human Reproduction (AHR) Regulation Viewed under the Lens of the Patient’s Experience

**DOI:** 10.3390/ijerph19159534

**Published:** 2022-08-03

**Authors:** Lauraine Ronan, Olivia McDermott, Mary Butler, Anna Trubetskaya

**Affiliations:** 1 College of Science & Engineering, National University of Ireland, H91 TK33 Galway, Ireland; lauraine.ronan@gmail.com; 2 College of Science, Atlantic Technological University, F91 YW50 Sligo, Ireland; butler.mary@itsligo.ie; 3 Department of Engineering, University of Limerick, V94 T9PX Limerick, Ireland; anna.trubetskaya@ul.ie

**Keywords:** assisted human reproduction treatment, IVF regulation, Ireland, in vitro fertilisation treatment

## Abstract

Assisted Human Reproduction (AHR) treatment is unregulated in Ireland, although it is practised there. Within Europe, Ireland is one of the only European countries without any form of AHR-specific regulation. This study aims to investigate the experiences and viewpoints of Irish women undergoing AHR treatments and establish if the lack of legislation is affecting these experiences. A quantitative survey was carried out on women undergoing AHR treatment in Irish clinics. Patients highlighted a lack of information in terms of end-to-end care and poor information around treatments and success rates. Key issues highlighted included unanticipated high treatment costs and add-on treatment costs, lack of financial support from the government, no redress process in the event of dissatisfaction, and generally an overall feeling of a lack of support both from the AHR clinics and the Irish government. This study offers a real-time view of the Irish AHR system from the patient’s experience of AHR and under the lens of the lack of a legislative system. In early 2022, the Irish government announced that it would adopt its bill around AHR treatment and that subsidies for AHR treatment are to come into effect, which will alleviate some financial pressures on patients. Further studies of the legislation carried out post implementation will provide more information about the impact of having a legislated AHR process on the patients.

## 1. Introduction

### 1.1. Ireland and AHR

Infertility affects approximately 3% of the worldwide population and has been diagnosed in 17–26 percent of couples of reproductive age [1]. Infertility is not only a medical condition, but it has social implications and can affect both the immediate couple’s relationship as well as relationships with friends and family members [2,3].

Since 1987, Ireland has been practising AHR, where currently, there are approximately ten main clinics that perform AHR treatments at the time of this study. Donor insemination is not offered, and few clinics offers donor eggs [4,5]. The Irish Medical Council has published guidance in relation to AHR for medical practitioners who are registered with them. This guidance refers to offering IVF only after a thorough investigation of other options, the use of qualified professionals with accredited facilities to provide services, and the governance and record keeping of donor programmes for traceability [6]. However, this guidance is not legally binding, medical professionals in the Irish AHR industry are not obliged to follow it [7], and not all AHR clinics register as medical practitioners. AHR Clinics are not obliged to submit details of treatments conducted in their facilities or publish any statistics on success rates. Currently, there is no authority in Ireland dedicated to AHR regulation. However, many experts agree that the lack of government regulation has increased clinic self-regulation [7,8].

Surrogacy is not addressed in Ireland [4]. The gap in legislation creates an uncertain legal situation related to the constitutional protection of the right to life of the unborn. There is also an undetermined legal status of embryos in vitro. The Irish Medical Council guidelines discuss surrogacy or how embryos are disposed of. However, the recently enacted bill will legislate for altruistic surrogacy arrangements and for surrogacy arrangements carried out entirely in Ireland, not to international arrangements [9].

### 1.2. AHR and Europe

Assisted Human Reproduction (AHR) has been increasing in healthcare in recent years. In Ireland alone, there are 6000 cycles of IVF treatment completed annually [10]. However, assisted Human Reproduction (AHR) treatment, although practised in Ireland, is not regulated there [1,11]. AHR is available privately, but not in any of the public hospitals in Ireland at the moment. This means that anyone that has wanted to get IVF treatment in Ireland has had to go through the private health system and often have to pay several thousand euros for one course of IVF treatment [12].

Ireland was alone in Europe as the only country without any form of AHR-specific regulation. At the commencement of this study, draft legislation had been with the Irish government since 2017, but it had not been introduced into law [13]. However, during the write up of this study, the Irish government announced, on 28th February 2022, that the Assisted Human Reproduction bill had been approved for publication into law [14].

Many Irish couples travel to European countries to access AHR services in more regulated and cost-effective jurisdictions. A previous study by some of this papers authors McDermott, Ronan and Butler, in early 2022, supported recommendations from many other studies and the Irish public that revisions be made to the draft legislation in order to meet Irish patient needs [15]. The study also concluded that as a benchmark and best practice model, a hybrid system similar to the UK’s and the Netherland’s AHR regulatory system would be ideal. The UK has a very efficient regulatory model in terms of organisation, practice, regulation and surveillance [16,17]. The Netherlands is considered a best model in terms of a financial support system [11]. A best practice in many countries is to have set price guidelines for the treatments, with a financial support system in place [18,19,20]. The linking of treatment for IVF to private health insurance is also favoured by governments in many countries. While many health insurance companies in Ireland offer IVF cover, not all treatments and clinics may be covered [21]. The AHR financial support system in the Netherlands has a basic obligatory mandated health insurance package that includes IVF, while IVF is free to women under 40 in the UK [22].

This study aims to follow up on the aforementioned legislative study by McDermott et al. in 2022 [15] and investigate the effects and experiences of Irish patients undergoing AHR. The authors will review how the absence of legislation (at the time of the research) may be affecting treatment and patient experiences.

This study aims to achieve the following:

- Investigate the experiences (positive and negative) of patients undergoing IVF/AHR within the Irish AHR system;
- Understand how Ireland’s current AHR regulation or lack of legislation has had an effect (if any) on the experiences of IVF/AHR patients.

## 2. Literature Review

Many AHR international protocols exist; some examples of these are in vitro fertilization (IVF), intracytoplasmic sperm injection (ISI), intrauterine insemination, timed sexual intercourse (TSI), Zentra functional biopsy (ZFB) and frozen embryo transfer (FET) which are very common procedures. During IVF, an egg is removed from the woman’s ovaries and fertilised with sperm in a laboratory [22]. Intracytoplasmic sperm injection (ICSI) involves injecting live sperm into a person’s eggs in a laboratory [23], while in intrauterine insemination, sperm that have been washed and concentrated are placed directly in the uterus during an ovulation cycle [24]. Zentra functional biopsy involves taking a biopsy of the womb lining and then analysing the genes of these tissues to pinpoint the ‘window of implantation’ when the endometrium is most receptive [25]. Frozen embryo transfer involves the transfer of an embryo into the uterus, in a process similar to a smear test [26].

Ireland, as a co-signatory to Directive 2004/23/EC Article 17, which sets out quality and safety standards for all treatments relating to human tissues or cells have via the Health Products Regulatory Authority (HPRA), implemented the Directive as they regulate laboratories and tissue management [4]. Under the new legislation passed in total by the Irish government in February 2022, the proposed regulatory authority called the Assisted Human Reproduction Regulatory Authority (AHRRA) will be set up and have administrative responsibility to supervise these practices and ensure compliance [8].

Many clinics offer adjunct treatments, and services can be offered to IVF patients [24]. These add-on treatments are offered and advertised by fertility clinics as enablers to improve patients’ chances of a successful outcome of their treatment. Prospective parents undertaking IVF treatment generally pay large amounts of money for treatment [11]. In the UK, the Human Fertilization and Embryology (HFEA) Act 1990 requires that patients in the UK be given any and all relevant information on any ‘add-on’ treatments, and they must give confirmed consent for these [25]. There is no such consideration in the Irish recently enacted “Assisted Human Reproduction Bill” for ‘add-on’ treatments. In comparison, in the UK, Clinics must provide open and honest information on the evidence that surrounds an add-on treatment [17]. There are a large number of these treatments on the market at the moment, with many void of any robust evidence [27]. Lensen et al. carried out a study in Australia among women having IVF in the previous 3 years, and they found that 82% had used one or more IVF add-on, with acupuncture, preimplantation genetic testing for aneuploidy and Chinese herbal medicine being the most common. Lensen et al. also commented on the limited information about the prevalence and types of use of these add-ons [28].

The Irish competent authority, or the HPRA, published the “*Guide to Regulatory Requirements for the Procurement of Human Tissues and Cells intended for Human Application*” in 2013. Applications must be made to HPRA by entities involved in the procurement (including testing, processing, preservation and storage) of human tissues and cells for authorisation to perform these activities. Clinics are required to complete the “*Application for Authorisation of a Tissue Establishment*” form and submit it to the HPRA. This guide is for guidance purposes and should be an interpretation of law or regulation [29]. HPRA has the authority to monitor these establishments. A list of all authorised establishments are published on the HPRA Blood and Tissue Establishment List–including the Irish fertility clinics [29].

The Commission Directive, 2006/17/EC, the Council Directive 2004/23/EC (standards for quality and safety for the donation, procurement, testing, processing, preservation, storage and distribution of human tissues and cells) and Commission Directive, 2006/86/EC (traceability requirements, notification of serious adverse reactions and events and certain technical requirements for the coding, processing, preservation, storage and distribution of human tissues and cells) were transposed into Irish legislation via the European Communities (Quality and Safety of Human Tissues and Cells) Regulations 2006 and 2007 (Statutory Instrument No.158 of 2006 and No. 598 of 2007) [29].

While the HPRA is the Irish competent authority for the enforcement and implementation of this legislation, they do not carry out audits on fertility clinics. The European Society of Human Reproduction and Embryology (ESHRE) has many guideline documents available on the ESHRE website [30]. Again, these are guidance’s only and clinics are not required to comply with them.

Under the new Irish legislation, a new AHR regulatory authority or AHRRA will be responsible for regulating treatments such as IVF, as well as the following: licensing and regulating domestic altruistic surrogacy; pre-implantation genetic diagnosis and other embryo-screening procedures; posthumous AHR, where pregnancy is achieved using the gamete or embryo of a deceased person, or an embryo created using the gametes of a deceased person [31].

## 3. Methodology

For this research, purposive sampling was deemed most appropriate for the research strategy [32]. Participants for the interviews were approached based on the fact that they had undergone AHR treatment(s) and had an experience with the Irish AHR system. This allowed in-depth data to be gathered on the personal experiences of the participants. The researchers contacted potential participants via a message about the study posted on social media forums for IVF participants. Generally, as people do not always advertise the fact that they are undertaking IVF treatment, this was deemed the best place to access potential participants. The final survey was disseminated to the relevant social media platforms and resulted in 50 responses.

### 3.1. Pilot Study Design

The first step in the development of the survey was to review the literature in relation to the research topic [33] and to answer the research questions, a questionnaire design process aided obtaining of reliable [34]. The questionnaire format will determine the type of questions asked and subsequently the types of data analysis [35].

The main information about the design of the questionnaire is presented in Table 1.

Data collection was an online questionnaire, which facilitates collecting data in a shorter period of time and reduces costs [36]. The questionnaire was developed using Google Forms and had two sections. The first section included the characterization of HEI (location, public or private type and size) and respondent details (position, years of experience and knowledge of the seven QC tools). The second section was dedicated to obtaining information about the use of the seven QC tools, critical success factors, barriers, challenges and benefits from the application of these tools. Questions related to barriers, challenges and benefits attempted to measure perceptions. The statements were evaluated using the seven-point Likert scale (1—Strongly disagree; 7—Strongly agree) as recommended by several authors (e.g., Sullivan and Artino, 2013; Hair et al., 2020). The survey questions are outlined in Table 2.

### 3.2. Data Analysis

The data were analysed using descriptive (graphs analysis). A reliability test has been performed to test the reliability of the questionnaire using Minitab^®^ 20 software. The analysis observed that the Cronbach alpha value of each set of IVF clinic selection factors is found to be satisfactory (as the values of Cronbach alpha >0.7). The calculated value of Cronbach alpha for each clinic selection factor is provided in Table 3.

## 4. Results

The patients who took part in the study were promised anonymity. The patient’s ages were in the range 33–46 years old (this demographic information was optional), and 100% of responses were from females. Even though many males participate in social media forums on IVF, it is mainly women who in the majority participate in social media related to IVF. Many studies have reviewed social media fertility support groups, and generally, females are very much in the majority [37]. Males were found in one study to be more “lurkers” than “posters” on these forums due to more insecurity as compared to women [38]. Ultimately, 50 people participated in the study.

### 4.1. The Clinics Involved

**Figure 1:**
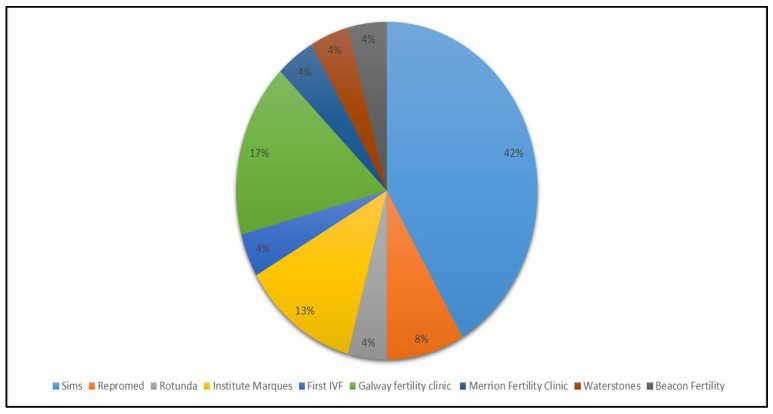
Clinics utilised by respondents who participated in this study.

Question one identified the clinic that the respondents attended. There were nine main fertility clinics operating in Ireland at the time of this study. Respondents were undergoing treatment in a large range of clinics as shown in Figure 1. Some respondents (20%) had attended more than one clinic. The top three clinics attended were SIMS (42%), which has more than one clinic in Ireland, the Galway fertility clinic (17%) and Institute Marques (formerly Clane clinic) (13%). The majority of Irish based fertility clinics are in the east of the country and based around the capital city, but this situation is changing annually. Some participants commented, “nearly all the clinics are in Dublin; there should be more services throughout the country”, and, “it is hard enough going through this treatment and taking time off work without having to travel (within Ireland) for it”.

All clinics offer a similar range of ART/IVF services based on a comparison of clinic websites reviewed by the authors. With the enactment of the new Irish Assisted Reproduction legislation, plans will be progressed to provide IVF via the public health system, which should, in theory, ensure a more geographic and financially accessible dispersion of treatment locations [39].

### 4.2. Types of Treatments Undertaken

**Figure 2:**
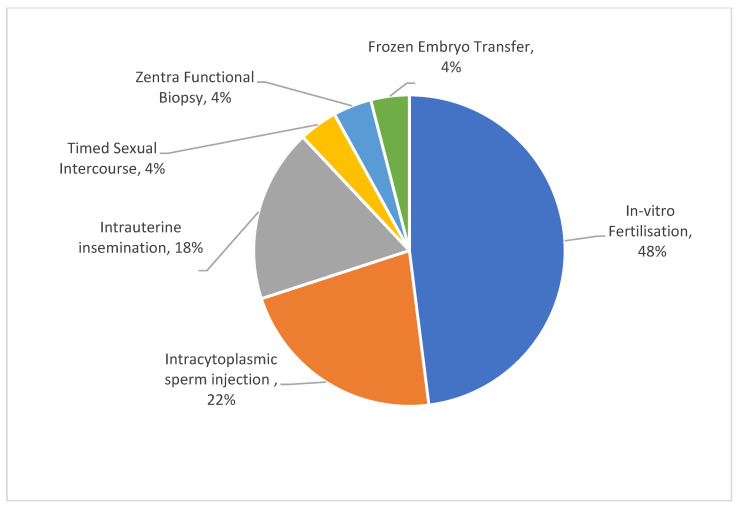
Types of treatments undertaken by respondents who participated in this study.

Question two ascertained the types of treatments that the respondents underwent. Some of the patients interviewed underwent multiple treatments and selected all of these in their responses. As shown in Figure 2, the most popular treatment was IVF, with 48% of the respondents having completed IVF treatment. The top three treatments undertaken were IVF (48%), Intracytoplasmic sperm injection (ICSI) (22%) and Intrauterine insemination (IUI) (18%). Other treatments, timed sexual intercourse (TSI), Zentra Functional Biopsy (ZFB), and frozen embryo transfer (FET) combined, comprised just 12% of the total %.

### 4.3. Reasons for Choosing a Particular Clinic

**Figure 3:**
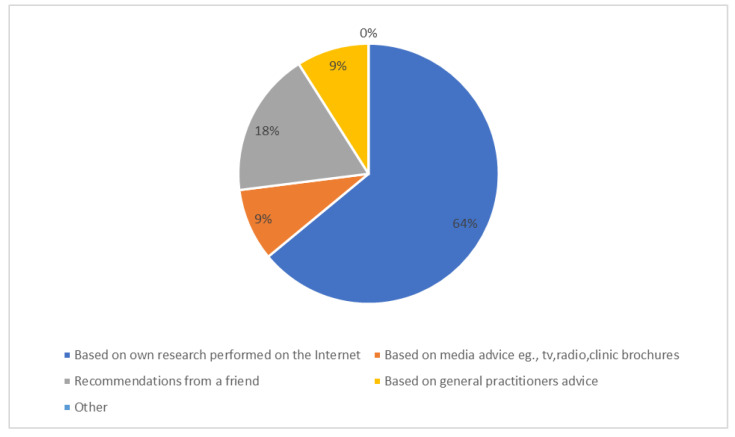
Reasons why the respondents in this study chose particular clinics.

The respondents were given five options for this question and a comments section. The results are shown in Figure 3. Overall, 64% based their clinic selection on their own research. The “Own research” category included social media forums as well as websites, and it has been found that social media forums around infertility are helpful in both helping process emotions around infertility and accessing information [37].

A much lower 18% of participants selected their clinics based on recommendations from friends. Wilkes et al. [40] in interviews with IVF participants, found that they generally avoided discussing their IVF with friends who had children but were more comfortable discussing if their friends had been through IVF.

A total of 9% made their selection based on the recommendations of their general practitioners (GP), and a further 9% selected their clinic based on exposure to media marketing, e.g., articles/radio/clinic brochures. Interestingly, the very low recommendations from GPs, at just under 10%, suggests a disconnect within the primary care health system and a lack of end-to-end treatment of patients with fertility issues. GPs have a very important and integral role in supporting patients undergoing IVF as part of a primary care system [41].

The new Irish legislation, with proposals to increase AHR service availability through the public health system, may increase knowledge and awareness of AHR services.

### 4.4. Amount of Money Spent on Treatments

Each patient was asked to state how much money they spent in total on their treatments. Results ranged from a minimum of EUR 3000 to a max of EUR 50,000. The average spent on AHR treatments by participants of the patient survey was just under EUR 15,000. Based on a review of the price ranges of fertility treatments on the website of these clinics by this papers authors, prices can start at as little as EUR 50 to EUR 250 for an initial consultation, with an average of EUR 4500 for IVF treatment. The high cost of IVF can be a significant factor in deciding to discontinue IVF treatment (should a live birth not occur) [42]. Medical insurance companies in Ireland vary in their policies regarding assisted reproductive treatments. They may cover consultation costs, and some may cover the cost of basic procedures, but the vast amount of funding is provided by the patient. Taxpayers can claim tax relief on medical expenses through the tax relief for medical expenses scheme, and Irish residents can avail of the Drug Payment Scheme. The scheme caps the amount a patient pays to their chosen pharmacy in a given month. The Irish Health Review Board has stated that the cost of one IVF cycle in an Irish private clinic starts from thousands of euros, including travel and drug expenditure [18]. The prices for AHR treatments vary, as do extra charges for add-on treatments. Many of these treatments have no proven safety or effectiveness data and are not regulated [43].

Based on the stages involved in these treatments and if multiple rounds of IVF are involved, costs can add up, and adjunct treatments can be used. One participant commented, “Clinics are only interested in money which has to be paid upfront regardless of treatments”. According to the Irish Minister for Health in February 2022, “The commencement of the new Irish AHR legislation will also allow us to progress the government’s plan to introduce the provision of advanced AHR treatment, including IVF, in the public health system” [14]. This will open up AHR to patients who cannot afford private AHR treatment, as AHR is not currently available in the Irish public healthcare system. The Irish Department of Health is planning to fund IVF services from 2023, with “regional fertility hubs” established before that [44].

### 4.5. Knowledge of Treatments Undergone

**Figure 4:**
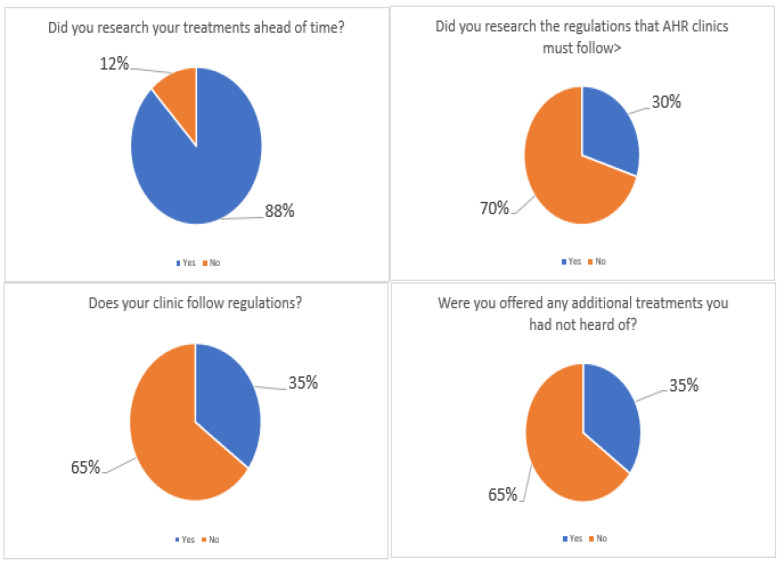
Questions about the respondents knowledge of the treatment and regulations around their AHR journey.

The next set of questions was related to patient knowledge of treatments and the regulations around them. The results show that 88% of participants researched the treatment they were going to have, but 70% did not research the regulations that the clinic must follow (Figure 4). In addition, 65% did not know if the clinic that they selected followed the regulations. The establishment of AHHRA provides some visibility to the legislative oversight of these clinics should patients wish to research this.

Similarly, 65% of the participants were offered treatments of which they had not heard. This would somewhat explain why the average cost for IVF treatment increased to about USD 15,000 and was higher than the average treatment package prices as advertised on the clinic’s websites. Comments from participants included, “while informed of the IVF process, I was not prepared for it emotionally”, “I would like a more individualised care package with a designated point of contact”, and, “There should be clearer information about treatments both medical and alternative”.

### 4.6. Awareness of Legislation around IVF/AHR

**Figure 5:**
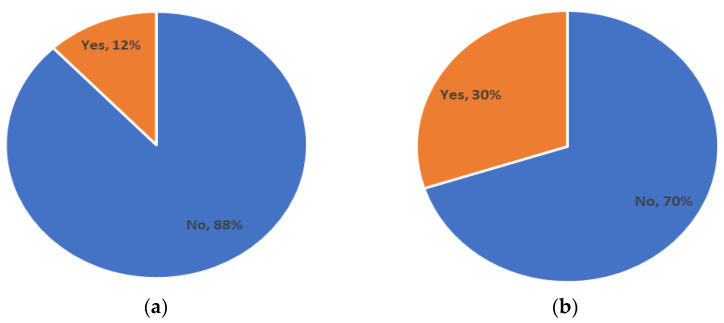
Questions around awareness of AHR legislation. (**a**) Awareness that Ireland has draft legislation to regulate AHR clinics. (**b**) Awareness that Ireland is one of the only European countries without regulated AHR clinics.

Participants were asked to identify if they were aware of the new bill on AHR currently with the Irish government for approval (at the time of the survey, the bill had not been passed into legislation). They were then asked if they were aware that Ireland is one of the only countries in the EU that does not have regulations surrounding AHR clinics. The results showed that 88% of participants were unaware of the proposed bill, while 70% were not aware that Ireland does not have existing AHR legislation (Figure 5).

In the comments section on the question in relation to the awareness of the bill, one participant stated, “I only cared about the end result, so this legislation would make no difference to my treatment”, and this theme was reflected in other comments entered. Other comments included, “this area needs to be regulated and recognised as a medical condition”, “Donor options and traceability are a concern for me”, and “access to supports, information, financial and psychological supports should be available and funded”. Based on the Assisted Human Reproduction legislation brought into law by the Irish government in February 2022 and building upon the Irish Children and Family Relationships Act 2015, the AHRRA will maintain a new National Surrogacy Register and the already-existing National Donor-Conceived Person Register. Thus, the area will be more regulated, and there will be traceability around donors. In relation to final supports as mentioned previously, the Irish government is set to put more financial supports in place in 2023.

### 4.7. Information around Treatment and Success Rates

A question was asked, “Do you think that you were well informed about treatment success rates, the procedures and expected results effectively by the clinic?”. It showed that 47% felt that they were well informed of the treatment’s success rate, procedures and expected results. However, 35% felt that they were not well informed, and a further 18% were not sure. Some participant comments in relation to this question were, “there should be more access to information”. Many commented that they felt “well informed” and “cared for”.

### 4.8. Criteria for Clinic Selection in Order of Importance

**Figure 6:**
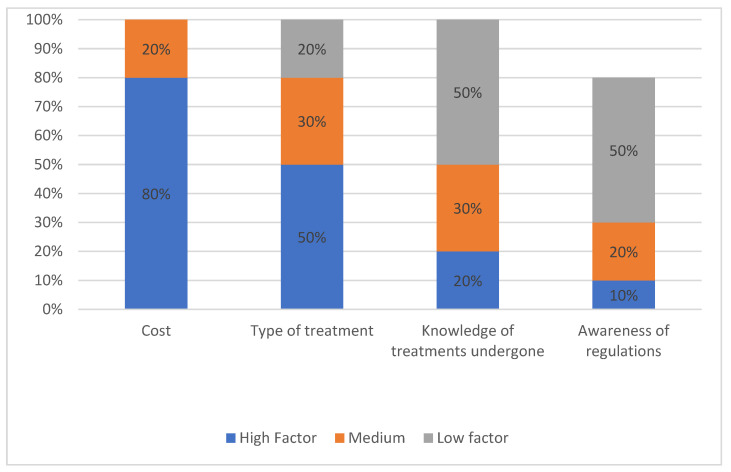
Criteria for Clinic selection in order of importance.

The participants were asked to rank the criteria for clinic selection (cost, type of treatment required, knowledge of treatments and awareness of regulations) in terms of whether they were of high, medium, or low importance to them (Figure 6).

Cost was a major criteria of high importance (80%) for selecting a clinic or treatment in that clinic, while the type of treatment required was the next highest factor of importance (50%). Knowledge of treatments undergone and awareness of regulations were not deemed high factors and were rated as low factors for election of clinics at 50% for both criteria. Note: not everyone answered the section on awareness of regulations.

Chi-square analysis was carried out to establish if there was a statistical relationship between the criteria for clinic selection. The chi-square value was 25.2 and was greater than the ranked value of 12.59, and thus, the null hypothesis was rejected, indicating a relationship exists between the criteria for clinic selection.

### 4.9. Improvement in Treatment

An open-ended question was asked to reinforce previous questions “Do you think there are any improvements that can be made to the fertility treatment process/journey?” The main results again reiterated previous themes that the participants felt “a lack of support”, “a lack of clarity”, and “feelings of neglect”. It was highlighted that the system was “open for abuse by clinics especially in pricing” and was “financially” and “mentally” exhausting. Patients highlighted that there was “not enough information” provided to them by the clinics and that “more care” and “advice” was needed on a “daily 24/7” and “post-procedure” basis. It was felt by many participants that this care “was not available”, that the patients were “not cared for”, and that the theme that “the clinics were only interested in money” was reiterated. While the new legislation may not even impact some of the aforementioned issues, the AHRRA regulation of AHR clinics will improve oversight and accountability. The move to offer AHR through the Irish public health system and the establishment of regional fertility hubs will, in effect, ease some of the psychological and financial stresses on patients.

### 4.10. Experiences of the Overall Process

The final question was again an open question, “How would you describe your experience in an AHR clinic overall?” The answers to this question were similar to the previous question. The themes were echoed again that patients felt that the experience was “extremely stressful”. The results again showed that patients felt like they were “just a number” in “a badly designed system in terms of proper care”. One patient noted that the AHR industry “exploits us based on the costs they charge and on our desire for a family and these clinics need regulating and the government needs to finance treatments”. It was noted again, as mentioned previously, that there is “a lack of clinics in the West of Ireland compared to the options available in the more populated capital city Dublin”. It was also noted that some patients had experience in clinics in Ireland and in other parts of Europe and described the European clinics as being “far superior”. The main theme that echoed through the results was the “expense” and the “lack of support”. Table 4 has highlighted some more specific responses which were a mix of positive, neutral or mixed and negative comments. Overall, the responses were more negative than positive in terms of participant experiences.

## 5. Discussions

This study set out to investigate the experiences of patients undergoing AHR within the Irish AHR system, with a view to understanding how much of a role or impact that Ireland’s then lack of legislation may be having on these experiences.

Different themes arose in this study around the experience of patients undergoing AHR, including lack of information and availability of treatments, high costs, and limited support in relation to AHR treatments (RQ1). While having regulations may not change the experiences of these participants, a more legislated process would benefit all (RQ2). Legislation was deemed positive by the majority of participants and “*not a bad thing*” although some stated that “*it wouldn’t affect them*” or “*change what we are going through at the end of the day*”. In their 2013 study on Irish public opinion on IVF, Walsh et al. found that a substantial number of the Irish public agreed that the Government of Ireland should introduce legislation covering AHR [7,45].

Legislated AHR treatment is the best practice around the world and can ensure a uniform and controlled service provision. International best practice also promotes that AHR treatments be at least partially funded by governments [11,15,30].

It should be noted that the new Irish legislation still is considered to have many gaps. An analysis of the Irish proposed (and now enacted) legislation by the Joint Committee on Health in 2019 recommended some changes which may bring Ireland more in line with other practices around AHR in European countries [46]. In addition to these recommendations, funding integrated into legislation, as in other countries, has been put forward as a best practice.

There is no visibility to provide insight or comparison into the different fertility clinics in Ireland. These clinics report their success rates in different ways and can offer add-on treatments without any scientific proof of their effectiveness. At the moment it can be difficult to compare one clinic with another without central, verified data sources on success rates of AHR with information on outcomes from AHR across all clinics in Ireland. While success rates can be published on individual clinic websites, De Geyter et al. (2018) found that only three Irish clinics reported their success rates to a European AHR registry [47]. The new Irish legislation will address this and provide oversight and transparency. The new proposed Irish legislation will introduce a dedicated, competent authority—the assisted human reproduction regulatory authority (AHHRA), which will provide regulatory oversight of Irish AHR. The objective of the AHHRA is to protect, promote and, as far as is practicable, ensure the health and wellbeing of children born as a result of assisted human reproduction, the intending parents, and other persons involved in the process [15].

Similar to the UK system, the AHHRA will grant licences and will have the power to revoke these licenses if clinics are not complying with the conditions outlined in the Act. The AHRRA will publish and maintain codes of practice giving guidance for the proper conduct of activities.

In summary, the findings of the study were mixed in terms of the experiences of the participants of the Irish AHR system. However, legislative oversight and review can only make the AHR process more transparent, controlled, accountable, more accessible to all and aid in financial support.

## 6. Conclusions

This research has discussed the experiences of patients undergoing AHR treatment in Ireland under the lens of the current situation with lack of legislation under Irish law. While having legislation around AHR may not impact many of the emotional and psychological stresses of undergoing AHR, having legislation can only enhance patient safety, access to treatments, rights to redress and information to ensure their rights.

The findings from this research will be useful to medical practitioners who wish to advise patients on AHR and members of the public who wish to avail of AHR services in terms of providing a reference and summary for them. As a research study, this paper should add to the growing number of literature citing the importance of legislation around these practices at a government level and informing government policy. The limitations of this study would be that it was solely based on the patient viewpoint, and the survey number was limited based on the availability of participants from social media support forums to the authors. There is an opportunity for further study in terms of the Irish IVF providers’ viewpoints and experiences of the Irish AHR system. Additionally, it is planned to conduct a follow-up study post legislative changes which have been approved by the Irish government.

## Figures and Tables

**Table 1:** Questionnaire design processes.

Survey Design Steps	Procedures
Select the variables/indicators to represent the concepts and measurement scale	The variables were identified in the literature review based on studies about AHR participants and regulatory environments.
Define target population and sampling frame	The aim is to carry out a pilot test for a study on the target population to investigate the experiences (positive and negative) of undergoing IVF/AHR within the Irish AHR system.
Determining question types, format, and sequence	The questionnaire was designed with open and closed questions and divided into two sections. The first one was related to the respondents’ profile and other demographic information. The second part was focused on the investigation of the experiences (positive and negative) of patients undergoing IVF/AHR within the Irish AHR system. Additionally, this section focused on factors affecting treatment selection, and choice of IVF centre as well as knowledge of legislation and rights.
Pretesting the questionnaire	The pre-testing was carried out with six people undergoing AHR as well as with two academics to cover both theoretical and practical aspects of AHR treatment in Ireland. The objective was for respondents to indicate difficulty in completing, responding or lack of questions or in the sequence of questions.
Report	The analysis of the questionnaire allowed preliminary analysis on the experiences of patients undergoing IVF/AHR within the Irish AHR system and an understanding of how Ireland’s current AHR regulation or lack of legislation has had an effect (if any) on the experiences of IVF/AHR patients.

**Table 2:** Survey Questions (non-demographic).

What clinic did you attend? Tick as appropriate *A list of all Irish clinics was provided*
What treatment did you have? Tick as appropriate or comment *A list of treatments was provided*
Why did you choose this clinic? Tick as appropriate *A list of reasons for choosing clinics was provided*
What research did you do? Tick as appropriate *A list of research types was provided*
Approximately how much have you spent in total on AHR treatment? *A range of costs was provided*
Did you research the treatment that you were going to have? *Yes*/*No*
Did you research any regulations that AHR clinics must follow? *Yes*/*No*
Do you know if your selected clinic follows these regulations? *Yes*/*No*
Please rank the following criteria for clinic selection (Cost, type of treatment required, knowledge of treatments and awareness of regulations) in terms of choosing a clinic as to whether they were of high, medium, or low importance to you?
Were you offered any additional treatments of which you had not heard? *Yes*/*No*
Are you aware that there is a proposed bill with the Irish government to regulate AHR clinics? *Yes*/*No*
If you have read or are aware of this bill, do you think it would have improved your treatment/experience had it been implemented? *Yes*/*No*
Are you aware that Ireland is one of the only EU countries that do not have regulations around AHR clinics? *Yes*/*No*
Do you think the laws do enough to protect AHR patients? *Yes*/*No*/*Do not know.*
Do you think you were well informed of the treatment success rate, procedure and expected results before you had your treatment? *Yes*/*No*/*Not sure*
Do you think there are any improvements or clarifications that need to be made immediately? *Please comment*
How would you describe your experience in an AHR clinic overall? *Please comment*

**Table 3:** Reliability test of clinic selection factors (Minitab Software).

IVF Clinic Selection Factors	Cronbach Alpha
Cost	0.852
Type of treatment	0.835
Knowledge of treatments undergone	0.773
Awareness of regulations	0.701

**Table 4:** Summary of some responses about participants’ experiences of the overall process.

*“Excellent experience, I have a 3 year old son as a result that I never would have had”*
*“Stressful”*
*“Has been more difficult with COVID-19 as I have not met my fertility doctor”*
*“Extremely expensive as a patient I was not given the holistic level of care needed”*
*“My experience of my current clinic in Spain has been far more superior than in Ireland”*
*“Overall good and I did have a son thanks to them”*
*“Really poor experience however the clinic I am currently with seems a lot better and I feel looked after and cared for as an individual”*
*“Lack of a case management approach, means the patient has to be to be their own case manager, overall, a stressful experience”*
*“A nerve wrecking experience and I am a nurse so feel I have some insight from a medical point of view, the experience would have been so much worse otherwise”*
*“Very reassuring and informative”*
*“Always found the clinic friendly and set us at ease”*
*“Hidden costs led us to feel unsure of our clinic as we paid a large bill and 2 months later got another one, very frustrating and upsetting as it so expensive “*
*“I have had a positive experience so far but still feel like a “rabbit in the headlights” as there is always something else I should have been aware of “*
*“OK but extremely stressful, lots of unnecessary waiting on results”*
*“Dismissive of me and not very investigative or informed. I had to point out information and facts out from my own research”*
*“I cannot fault my clinic”*
*“Communication from the clinic can be poor at times”*
*“The clinic staff were generally nice but you were very much a number”*

## Data Availability

Not applicable.
